# Causal Attributions in an Australian Aboriginal Family With Marfan Syndrome: A Qualitative Study

**DOI:** 10.3389/fgene.2020.00461

**Published:** 2020-05-07

**Authors:** Aideen M. McInerney-Leo, Jennifer West, Bettina Meiser, Malcolm West, Matthew A. Brown, Emma Duncan

**Affiliations:** ^1^Dermatology Research Centre, The University of Queensland Diamantina Institute, The University of Queensland, Brisbane, QLD, Australia; ^2^Prince Charles Hospital Clinical Unit, School of Clinical Medicine, The University of Queensland, Brisbane, QLD, Australia; ^3^Prince of Wales Clinical School, University of New South Wales, Sydney, NSW, Australia; ^4^Translational Genomics Group, Institute of Health and Biomedical Innovation, Queensland University of Technology, Brisbane, QLD, Australia; ^5^Guy’s and St Thomas’ NHS Foundation Trust and King’s College London NIHR Biomedical Research Centre, London, United Kingdom; ^6^Department of Endocrinology, James Mayne Building, Royal Brisbane and Women’s Hospital, Herston, QLD, Australia

**Keywords:** causal attribution, Marfan syndrome, genetics, Aboriginal Australian, qualitative, psychosocial

## Abstract

Causal attributions are important determinants of how health threats are processed and affect health-related behaviors. To date, there has been no research on causal attributions in genetic conditions in Aboriginal Australians. Forty members of a large Aboriginal Australian family with Marfan syndrome (MFS) were invited to participate in an ethically approved study exploring causal attributions, including perceived causes of phenotypic variability within the family. Eighteen individuals consented to conduct semi-structured qualitative interviews, which were recorded, transcribed verbatim and analyzed thematically. Most participants knew that MFS was genetic, but there were diverse theories about inheritance, including beliefs that it skipped generations, was affected by birth order and/or gender, and that it co-occurred with inheritance of blue eyes within this family. The mutation was thought to have been inherited from British settlers and initially triggered by disease or diet. Factors believed to modify disease severity included other genes and lifestyle factors, particularly alcohol and substance abuse and stress. Generally, this family did not endorse “blaming” chance or a higher power for phenotypic variability, though some felt that the spirits or a deity may have played a role. In conclusion, although participants knew MFS was a genetic condition, many speculated about the role of non-genetic causes in initiating the original mutation; and the gene-environment interaction was thought to affect severity. This study demonstrates a successful approach for exploring causal attributions in other genetic conditions in First Australians.

## Introduction

Marfan syndrome (MFS) is a connective tissue disorder, affecting 2–3/10,000 individuals (Judge and Dietz, 2005). MFS is an autosomal dominant condition caused by mutations in *FBN1* (Lee et al., 1991). The main clinical features are aortic root dilatation and ectopia lentis (lens dislocation); other features include increased arm span, pectus excavatum or carinatum (sunken or bulging breastbone, respectively), scoliosis, joint laxity, myopia, spontaneous pneumothorax, and mitral valve prolapse (Marfan, 1896; Loeys et al., 2010). Aortic root dilatation in MFS patients can lead to aortic dissection and sudden death, though surgical and/or medical intervention can reduce the otherwise high mortality of this condition (Brooke et al., 2008). Lens dislocation occurs in the majority of MFS patients, and can occur after minimal or absent trauma, compromising visual acuity (Hindle and Crawford, 1969).

There is considerable intra- and inter-familial clinical variability in MFS, suggesting genetic and/or environmental modifiers (Dietz et al., 1992; Dietz and Pyeritz, 1995). Potential genetic modifiers include the expression of the non-mutated copy of *FBN1* (i.e., inherited from the unaffected parent) (Hutchinson et al., 2003; Aubart et al., 2015) and/or variants in related genes (Fernandes et al., 2016). Environmental factors likely to affect risk of aortic dilatation and/or dissection include hypertension, chest trauma, illicit drugs, and pregnancy (Hirst et al., 1958; Larson and Edwards, 1984; Nistri et al., 1999; Lange and Hillis, 2001).

Illness representations (i.e., people’s perceptions of, and beliefs about, an illness) affect how health threats are processed as well as subsequent health-related behaviors (Leventhal, 1970; Leventhal et al., 1997, 1998, 2003). Illness representations are not static but change in reaction to life experiences, including experiences of illness and medical care personally and/or in family and friends, media exposure, and cultural beliefs (Leventhal et al., 2003). The component of illness perceptions most strongly pertaining to genetics is causal attribution, the process of analyzing a situation and identifying the reason that a major event occurred (Leventhal et al., 1997). After receiving a diagnosis of a serious medical condition, 70–95% of people strive to identify and attribute an explanation (Turnquist et al., 1988), in an effort to restore the belief that the world is coherent, cohesive and predictable (Abramson et al., 1978; Taylor et al., 1984; Affleck et al., 1987). As with illness perceptions generally, causal attributions may change over time (Hunt et al., 1989).

Shiloh et al. (2002) asked participants (predominately university students) to group possible causes of disease by perceived similarity. Three overarching categories emerged: environmental, behavioral, and “hidden.” Genetics was assigned to the hidden category, along with mystical and psychosocial causes. The hidden category was perceived to be the least controllable. This is troubling, as control, or at least perceived personal control (Berkenstadt et al., 1999), is important in coping (Scharloo et al., 1998) and affects health behaviors (e.g., medication adherence, clinic attendance, etc.). However, elsewhere Shiloh points out that the perceived lack of control is also associated with decreased blame, which in turn aids coping with genetic conditions (Shiloh, 2006). Shiloh postulates that individuals attending genetic counseling are constantly balancing the desire to decrease blame with that of increasing control (Shiloh, 2006).

Few studies are available on causal attributions in genetic conditions generally and only one addressing causal attributions in MFS specifically (Peters et al., 2001). Peters et al.’s (2001) study, which was carried out in the United States and assessed 174 people with MFS, found that aortic dissection, joint pain, and depressive symptoms were all negatively associated with a belief that MFS was a curable and/or controllable condition. The majority felt they had low personal control over their illness and endorsed heredity as the cause, while half believed that chance played a role. These attributions seemed consistent with the presence or absence of a family history of MFS, in that individuals with a positive family history were more likely to select heredity, whereas those with no family history (i.e., in whom MFS resulted from a new mutation), were more likely to select chance (Peters et al., 2001). This study also demonstrated that it was possible to hold multiple, mutually exclusive causal attributions. Notably, this study predominantly focused on factors initially causing disease, rather than those modifying disease severity.

To the best of our knowledge, no psychosocial research has been reported in Aboriginal and Torres Strait Islander Australians pertaining to causal attributions in genetic conditions. Most available psychosocial research in First Australians has centered on cancer care and support provision, showing that cancer was sometimes perceived to be caused by “payback” and, in those instances, Aboriginal people were less likely to seek assistance for pain management (McGrath, 2006b). The idea of relocating for cancer care was frightening and encompassed a fear of leaving home, cultural alienation, disempowerment, losing support, and being in a hospital environment (McGrath, 2006a, 2007; McGrath et al., 2007).

In summary, there is little published regarding causal attributions in MFS and nothing exploring individual interpretation of phenotypic variability within a family (despite all family members sharing a common mutation). Further, there have been no previous studies of causal attributions in any genetic condition in Aboriginal Australians. Here, we report causal attributions and individual interpretation of familial phenotypic variability by affected and unaffected family members of a large Indigenous Australian family with MFS.

## Materials and Methods

### Study Design

This study was approved by the University of Queensland Human Research Ethics Committee (UQ #2014001158). Affected and unaffected family members (including spouses/parents), who had previously participated in a genetics study of MFS (Summers et al., 2004) were invited to participate. Individuals were invited to participate either by mail or in person (by JW, a cardiac nurse known to the family). All individuals invited to participate were given an invitation packet, which included a letter of invitation, an information sheet, consent form, an opt-in/opt-out response sheet and a stamped addressed return envelope. Those who had not responded after 2 weeks were telephoned to address any questions or concerns. Additional invitation packets were sent if initial sets had been misplaced or had not arrived. Finally, after some participants had been interviewed, they were encouraged to contact other family members to “vouch” for the study; this included both previously invited individuals and other members of the family not previously contacted. Consented participants were interviewed in person or by telephone, depending on their preference, and at a time of their choosing.

A qualitative methodology was chosen as the optimal initial exploration of this topic. In-depth, semi-structured interviews were conducted by AM-L exploring participants’ perceptions of MFS, including its challenges, benefits and controllability, and participants’ perceptions of causal attribution both for MFS *per se* and its clinical variability. Sampling was discontinued when no more individuals were willing to participate; additionally, at this point data saturation had been reached (Denzin and Lincoln, 1994).

### Data Analysis

Interview recordings were transcribed verbatim. The conceptual framework of Miles and Huberman (1994) was used to guide analysis. Analysis was both deductive (utilizing theoretical frameworks generated from past research) and inductive (identifying emergent themes and patterns pertaining to each category). Transcripts were coded by an accredited genetic counselor and experienced qualitative researcher (AM-L) to assign themes and concepts based on the topics covered in the semi-structured interview guide. In this manner a preliminary codebook was developed. Subsequently all transcripts were re-coded, this time subdividing thematic categories (nodes) into more fine-grained sub-categories (e.g., lifestyle was subdivided into exercise, in turn subdivided into high impact exercise, inadequate exercise etc.). These sub-nodes were further refined or merged as needed and transcripts reanalyzed until no new codes were required; and an advanced codebook was generated. All transcripts were then imported into QSR International’s NVIVO 11 qualitative data analysis software (Richards, 2005), and transcripts were coded using accepted coding techniques (Coffey and Atkinson, 1996). As a strategy to reduce bias and enhance validity (also referred to as credibility in qualitative research) (Miles and Huberman, 1994), four transcripts were coded independently by BM, a research psychologist with expertise in qualitative research methodology and psychosocial aspects of genetics. Codes were reviewed and reconciled. All themes were evaluated against participants’ disease status [i.e., whether they had a personal diagnosis (affected) versus no personal diagnosis (unaffected) with MFS]. Each illustrative quotation in the results section is followed by an ID number and a code denoting the disease status of the respondents, i.e., unaffected (UA) or affected (AF) individuals. In the interest of confidentiality, unaffected siblings/offspring are not distinguished from unaffected spouses/parents.

## Results

Letters of invitation were sent initially to 27 members of a large Indigenous Australian family with MFS located in two rural towns. Six returned the response sheet agreeing to participate. One letter was returned due to an incorrect address. Follow-up phone calls identified eight additional individuals willing to participate, and five individuals were invited in person at an outreach clinic. Thus, a total of 40 individuals were invited, of whom 18 participated in an interview, giving a participation rate overall of 45%.

From February to October 2015, 13 women and 5 men ranging in age from ∼20 to 70 years were interviewed (specific ages not given to protect confidentiality). Mean interview length was 23 min (range: 10–46 min). Of the 18 participants, 8 had MFS, 6 were unaffected family members, and 4 were spouses and/or parents of affected individuals. Fifteen of the 18 participants were parents, and of these 10 had affected children. All had experienced the death of at least one first- or second-degree relative from complications of MFS. Specific clinical details of affected individuals are not provided in order to protect confidentiality. However, all affected individuals met the Revised Ghent Criteria for MFS (Loeys et al., 2010), including lens dislocation, and their diagnosis had been genetically confirmed. Five of the eight individuals with MFS were employed, employment status was not collected on unaffected family members. None of the participants had a university degree.

### Factors Perceived to Cause Marfan Syndrome

Almost all participants (16/18) identified that MFS was heritable. While some stated a possible chromosomal “imbalance,” the majority specifically mentioned genes.

*I think it’s just dicky genes*. *ID 6* (*AF*)

#### Eye Color

It was also suggested that MFS was associated with eye color, such that those with blue eyes had the condition and those with brown eyes did not.

*Like my mum, she knew* [*Name*] *was going to have it because he had blue eyes. She knew straight up. She said he’s going to have eye problems. ID 7* (*UA*)

#### Skipping Generations, Birth Order and/or Gender

A few individuals commented that they had either observed or were concerned about MFS skipping generations.

*Um, because of our son and it’s something that I want him to be aware of because there’s you know there’s that chance that it’s going to be passed on to his children. And possibly even* (*my other child’s*) *may – I’m not sure if it skips a gene or whatever, yeah I’m not too sure on that. ID 15* (*UA*)

A few people felt that birth order correlated with the likelihood of having MFS or the severity of the disease, while two suggested that males were more likely to be affected than females.

*From what I have seen in, just in my family, the eldest child always has the same features and always has the same little idiosyncrasies*…*.so it seemed to be the first child of everyone, and then after that the severity got worse*…*especially if it’s a boy like.*…*.I think it’s because the boys live harder or I don’t know and they, their bodies grow big, bigger than ours, I don’t know, but they seem to die very young in our family. ID 2* (*AF*)

#### Where did the Mutation Originate?

Although most participants acknowledged that the condition was genetic, a third were perplexed about the origin of the mutation within the family. The responses indicated that the origin of the mutation had clearly been a topic of considerable thought and discussion.

*And that dates back to then and we still don’t know where we actually got it. We know we got it off our grandfather but where did he get it from, you know? Like where did it actually come from in the first place? ID 4* (*AF*)

Some participants did not believe that a mutation was spontaneous, but rather something and/or someone had introduced it into the family. Four people expressed the belief that it was not a condition which originally occurred in Aboriginal Australians but that it was introduced by the English or Europeans when they came to Australia. As this family had a couple of known ancestors from Britain, a few surmised that the condition was introduced into the family via one of these ancestors.

…*that’s where I, I think it would come from anyway because we didn’t have these problems in this country before that at all. ID 2* (*AF*)

Some felt that knowing that it originated with Europeans still did not explain the cause of the original mutation and some believed that environmental factors such as disease or diet triggered the mutagenesis.

*Uh well I was just under the belief that it was brought into this country, into my family, through the uh English um who had you know picked up scurvy and syphilis on board and brought it over here and obviously impregnated my lineage along that family tree*…*.that were brought to this country via um, the English who had syphilis and that was where they, um, that was you know when the fetus was in there, that was where the chromosomes started breaking down. ID 2* (*AF*)

*It could go back to the parents and grandparents and the breeding. Back to the things uh food that they ate probably years ago*…. *You never know back years ago it couldn’t have happened*…*.older people hadn’t been hygienic years ago eh? Could have contracted some sort of germ or something. ID 12* (*UA*)

### Factors Influencing Phenotypic Severity

Participants were asked whether they had any theories to explain the clinical variability within the family. About half responded that they didn’t know, while the remainder felt it might be simply the way people were born, lifestyle, or additional genes.

Interviewees were then asked whether and how each of the following factors contributed to phenotypic severity: lifestyle; stress, emotions or worries; germs, pollution or environmental factors; chance or bad luck; spirituality; and other genes.

#### Born This Way

The most common theory as to why there were phenotypic differences within the family centered on the belief that “everyone’s different” (ID 4, AF) and the acceptance that one is “born with it” (ID 5, UA). This response was volunteered by about half the participants.

*I think to me it affects everyone in different ways. Everyone’s different. Like someone can have the heart problem and not have, um, the skeletal feature problems*…*.that’s, to me that’s how I think how it affects different people, just – it just affects different people in different ways. ID 17* (*UA*)

#### Lifestyle

Most participants repeatedly stated that lifestyle affected severity. Lifestyle factors thought to play a role included alcohol, physical activity (overexertion or lack of exercise), drugs, diet, self-care (good self-care or lack thereof) and smoking.

##### Alcohol and drugs

Alcohol and drugs were repeatedly blamed for exacerbating the cardiac symptoms of MFS, and participants drew direct relationships between abuse of these and aortic dissection. Affected and unaffected individuals alike suspected that substance abuse moderated disease severity.

*Well I think it’s lifestyle um you know like uh for example I know* [*name*], *he died when I was* [*age*], *uh he lived pretty hard, he was you know a heavy binge drinker, um he did drugs, he played football you know high-contact sport um and the same with* [*name*] *you know.er he smoked, you know they all smoked and they played rugby league and you know high contact sport and fighting and stuff like that. ID 2* (*AF*)

*Oh well because – well if I wasn’t drinking now and after having a heart operation like, I’d probably be dead by now because alcohol and Marfan syndrome heart, they just don’t mix. ID 4* (*AF*)

##### Physical activity, diet and self-care

Participants seemed aware of the importance of staying healthy and taking care of their bodies when affected with MFS, specifically mentioning the role of exercise, diet, and self-care.

In terms of exercise, half of participants spoke of the challenge of finding the right balance for physical activity. The majority perceived danger from contact and high-impact sports. However, many believed it was important to stay active in order to stay healthy.

*Well yeah, um, if you can do – if you do the wrong exercise that can put strain on your body and strain on your heart and make it, you know, and make it worse. ID 17* (*AF*)

A healthy diet, specifically the avoidance of unhealthy food, was mentioned as important in maintaining physical health and an appropriate weight.

*Probably just your diet. You’ve got to um, like I said you’ve got to watch what you eat and then um keep it under control*… *don’t become too overweight and um because I think the more unhealthy and unfit you become, the easier it is to um, the easier it is for these things to take effect. ID 11* (*AF*)

Taking care of oneself also included adhering to medication and attending medical appointments.

#### Stress, Emotion or Worries

Although a few participants stated that stress, emotions or worries did not affect the severity of the disease, the majority felt that it did. Those who elaborated, explained that stress put additional strain on the heart and/or body.

Of note, participants articulated that the financial stress associated with the disease or the stress of not being able to work could also be deleterious.

*You know they don’t look after themselves very well, they don’t have, you know they’ve got financial stresses and you know all the things that kill normal people like you know not being financially sound and not having full-time jobs and stuff like that, that’s got to be stressful for people and put strain on the heart. ID 2* (*AF*)

#### Other Genes

Most participants thought that other genes could affect severity. One participant knew other individuals affected with a genetic neurodegenerative disorder, and she postulated that there could be an additive effect if one inherited both MFS and this neurological condition.

*Um, I do, yeah I do. I do think if someone had another – another genetic disorder, I think it could affect, it could affect Marfan’s in a different way. ID 17* (*AF*)

Some participants believed that the genes of the unaffected parent might modulate the severity of the disorder in their offspring, though they emphasized that this was difficult to assess ahead of time.

*I suppose you could have genes there that mixed together could cause, like ordinary genes that might react with the Marfan genes. So yeah, and you don’t know what genes you’ve got unless you’re a doctor and you’re looking under a microscope or something. ID 9* (*UA*)

#### Germs, Pollution or Other Environmental Factors

Respondents were divided as to whether germs, pollution and/or environmental factors affected the severity of the illness, with about half believing they did and about half believing that they did not. Pollution and chemicals were seen as potentially disease-causing in general and therefore could be a risk factor for MFS severity specifically.

*You know pollution has a large effect on a lot of genetic diseases you know like, I don’t know, cancer and stuff like that*… *you know pollutions and stuff, do release those germs or, you know, make them worse. ID 15* (*UA*)

#### Chance or Bad Luck

The majority of interviewees rejected the idea that chance or bad luck played a role in the severity of the illness.

*I don’t think it’s chance or bad luck. If they’re born with it, you’ve got it. ID 9* (*UA*)

Paradoxically, while disavowing chance, some participants used language suggestive of the potential role of luck.

*Bad luck, hmm. No. I think it’s just one in a million. So you just can’t say it’s a chance or bad luck thing. I think it’s just a one in a million, you know. ID 7* (*UA*)

However, a few felt that luck could have played a role. The majority spoke about chance and luck in terms of the probability of inheriting MFS in the first place rather than luck or chance affecting the severity.

*Yeah, yeah I reckon because not every single one of us has got it*… *You know what I mean like if it was bad luck everyone would have it*…*.Yeah well I reckon that, yeah. Some people have got um other diseases like that runs through their family and we just got – we just unlucky enough to have just Marfan disease that runs through our bloodline. ID 5* (*UA*)

One individual did mention luck or chance in conjunction with severity and, in doing so, referred to the gene contribution of the unaffected parent.

*If I can say I’m lucky and it’s just by chance that* [*names*] *got the worst part of the genes then yeah I think from my mother. ID 16* (*UA*)

#### Spirituality

The majority did not feel that spirituality altered disease severity. A couple of individuals rejected the idea that faith or spirituality could modulate phenotype, as they didn’t think it was fair to implicate God.

*But I look at it this way, you can’t blame God for what happens, you know. If you’re dealt something in life you deal with it or you try to, you know? ID 8* (*UA*)

*I’m not sure, just suppose if you’re born with it you’re born with it I guess but everyone’s, I don’t know, I’m a big believer in God tarnishes everyone with a different brush, you know what I mean, like can’t make us all look the same, everyone’s – people, everyone’s different in the world. ID 5* (*UA*)

One individual specifically stated that belief could not remove the disease from the body.

*For me, I’m more of a realist. I believe, like I’ve got it um and I don’t think um whether or not you, what your belief is going to make it any better or any worse because it’s one of these little nasty things that once it’s there, unfortunately I don’t think we can get rid of it out of your body. ID 11* (*AF*)

However, a few thought that spirituality could play a role and had very different theories. One person believed that the disease itself could have been caused by upsetting the spirits.

*Hmm something could have happened years ago, spirits didn’t agree with, or agree with or didn’t you know, never know. ID 12* (*UA*)

Another believed that the spirits of deceased relatives were watching over those who were affected today and protecting them.

*My grandfather had it, great grandfather. I’d say he’d be, you know, looking over them and stuff, all the family members. ID 10* (*UA*)

Another accepted that God sent challenges to everyone, and that MFS was just the challenge in their family.

*Yeah just tarred our family with the Marfan*…. *But yeah, no, it’s all good. It’s just a hurdle that God threw us, threw at us I reckon. ID 5* (*UA*)

Finally, one participant felt that spirituality could affect severity as spiritual people would want to be on the land, which was remotely located and thus far removed from services.

*Um maybe you know like cultural and spiritually you know with my family living remotely they want to be connected to land so they want to stay out there*…*so you know it sort of blocks them off from getting um help and getting services and getting fresh food and lifestyle choices you know having better choices I guess available to them because they want to be connected to the land, to the culture. ID 2* (*AF*)

## Discussion

This study is the first to explore causal attributions for a genetic condition in an Aboriginal Australian family. Interviews with eighteen affected and unaffected individuals examined the perceived cause of MFS in the family and found that though the majority believed that the condition was genetic, they were less clear about inheritance, suspecting that the condition could skip generations, and that both birth order and gender affected the probability of having MFS. Environmental factors, such as infections and poor diet, were thought to explain the origin of the initial mutation, which was believed to have been introduced by the British. When asked about the factors which were affecting the severity of the disease in affected individuals, lifestyle and other genes were most commonly discussed possibilities. This family did not ascribe to the belief that God or a spiritual entity caused or moderated the severity of their condition.

Sixteen of the eighteen participants knew that MFS was a genetic condition. This high percentage is unsurprising given that all symptomatic family members were offered genetic testing and counseling through the regional clinical genetics service 5 to 10 years prior. Nonetheless, this is consistent with a previous quantitative study of causal attribution in a MFS support group cohort, which found that 74% selected heredity as a cause (Peters et al., 2001). Causal attributions have been previously explored in genetic conditions generally; and a recurrent theme is the belief that physical features co-segregate with genetic conditions, i.e., a parent would “determine” that a child would or wouldn’t develop a familial condition based on their physical resemblance to another known affected family member (Kessler and Bloch, 1989; Huggins et al., 1992; Emslie et al., 2003; Shaw and Hurst, 2008; Klitzman, 2010). Psychologically, this “preselection” enhances coping by reducing ambiguity and uncertainty (Kessler and Bloch, 1989; Klitzman, 2010).

When the theme of eye color predicting affection status initially emerged from the data, it was tempting to view this as another example of genetic preselection. However, the gene associated with blue eye color, *OCA2* (Duffy et al., 2007) is located at 15q13.1, 30cM proximally to *FBN1* (15q21.1); linkage analysis in this family (not shown here) demonstrated that these loci co-segregate in this family. In Caucasian populations, the OCA2 locus is autosomal recessive and thus, its co-segregation with a dominant trait would mean that it was unreliable as a marker for disease. We do not have these data for Aboriginal Australians, and it is therefore, possible that these comments indicate that family members have astutely observed that blue eyes are a marker for MFS in this family. Of note, other Marfanoid syndromes, such as Loeys-Dietz, are associated with blue eyes, so modifier loci in related genes in the same pathway may be affecting eye color in affected members of this family.

The concept that genetic conditions “skip” generations is a widely held belief in lay populations (Richards, 1996; Emslie et al., 2003) and may at least in part be attributable to X-linked inheritance, incomplete penetrance, recessive diseases and traits (e.g., red hair), and transmission of translocations within families. Severity of MFS can vary significantly within families, with some people having ocular and/or cardiac involvement at an early age and others being more mildly affected (Dietz et al., 1992). However, it never “skips” generations. There are three possible interpretations for such a belief in this family. Firstly, participants may have been referring to severity of symptoms (i.e., variable expressivity) rather than the presence of the disease (i.e., variable penetrance). Secondly, in one branch of this particular family there are some individuals with isolated dilated aorta in the absence of MFS or an *FBN1* mutation; this would certainly add confusion to this family’s understanding of how MFS is inherited. A third explanation is that a belief that a condition could skip generations allowed people hope when choosing to have children, thus enabling them to engage in “reproductive roulette” (Lippman-Hand and Fraser, 1979).

Birth order does not determine disease status for MFS. This misconception has been reported previously in other conditions (Fanos and Johnson, 1995; Richards, 1996; Hunt et al., 2002). Such beliefs may be a psychological defense mechanism used to cope with the arbitrary nature of an inherited genetic condition (Richards, 1996), given the 50/50 likelihood of any particular allele being passed onto offspring. Although gender *per se* is not associated with MFS status, aortic dissection occurs at an earlier age in males with MFS compared to females (Murdoch et al., 1972).

People were perplexed by the origin of the mutation and speculated that it had been introduced into the family by British ancestors, who may themselves have acquired the mutation through disease or poor diet, particularly in pregnancy. There are a number of psychosocial reasons as to why this Indigenous family might believe that MFS had been introduced by Europeans. British colonization introduced many infectious diseases (including syphilis, tuberculosis, influenza, and measles) to the Indigenous population of Australia (Dowling, 1997). Australian Aboriginal Peoples also believe that cancer was brought to Australia by the “white man” (Prior, 2006, pg 27). Furthermore, as MFS segregates with blue eyes in this family, this may have reinforced the belief that it was originally a “British” disease.

The belief that consumption of certain foods during pregnancy causes congenital conditions is common, especially in certain ethnocultural groups (Cohen et al., 1998). For example, cleft lip is believed to be caused by the consumption of rabbit (hare) (Cheng, 1990), café au lait marks a consequence of fish consumption (Shaw and Hurst, 2008) and ectrodactyly (split hand and foot) a penchant for crab during pregnancy (Abad et al., 2014). Although exposure to certain toxins in pregnancy can undoubtedly result in congenital abnormalities, there is no association with such toxins inducing heritable mutations. However, holding such beliefs can promote a sense of personal control in subsequent pregnancies (Shiloh, 2006).

When asked to speculate as to factors influencing disease severity, most respondents replied that it was just the way people were born or simply because “everyone is different.” Previous qualitative research on Aboriginal Australians with cancer has shown that fatalism is common (Prior, 2005, 2006, 2009; Shahid et al., 2009). On considering reactions in this family on this point, interviews were closely evaluated but no language or tone was found suggestive of fatalism or resignation. A “what will be, will be” philosophy most accurately describes their reaction. Evaluated against the categories proposed by Shiloh et al. (2002) it appears that this best fits into the “mystical” category, which in turn belongs to the broader category of “hidden causes.” Hidden causes are associated with high levels of uncertainty and low levels of control (Shiloh et al., 2002), which would appear to bode poorly for coping in this family, given that optimal coping traditionally involves limiting uncertainty and regaining control (Leventhal et al., 1997). However, Shiloh also points out that control can be “associated with undesirable feelings of responsibility and self-blame” (Shiloh, 2006, pg 331). Thus participants choosing the “hidden causes” explanation may be absolving themselves of any guilt associated with disease severity. Interestingly, when causal attribution in cancer was qualitatively explored in an Australian Aboriginal sample, the etiology of cancer was often ascribed to payback for the perceived misdeeds of the affected individual, again falling into the “mysterious” category, which researchers felt promoted acceptance and coping (McGrath et al., 2006).

Lifestyle was the second most commonly endorsed modifier of phenotypic severity, with drug and alcohol abuse being the most frequently named. Drug use, particularly amphetamines are known risk factors for aortic aneurysms (Westover and Nakonezny, 2010). The relationship between alcohol consumption and aortic dissection risk has been predominantly studied in abdominal aortic aneurysms and there appears to be a complex relationship, where low to moderate consumption is associated with having a reduced risk, as compared to non-drinkers, but higher levels of consumption are associated with increased risk (Wong et al., 2007; Green et al., 2014). Smoking is a well-known risk factor for aortic dissection (Wong et al., 2007), but this was not mentioned as frequently as other lifestyle factors. This may reflect the fact that 56% of Aboriginal Australian Peoples located in remote communities are smokers (Australian Bureau of Statistics, 2015), and suggests that this is less likely to be perceived as a negative health behavior. However, in a survey study Aboriginal Australian smokers held similar views about the negative impacts of smoking compared to smokers in the general Australian population (Nicholson et al., 2015). This family’s concern about the deleterious effect of high impact sports on aortic dilation are consistent with medical advice as this is a known risk factor for aortic dilatation in MFS (Ammash et al., 2008). Finally, diet and moderate exercise were seen as factors which could lower the risk of aortic dissection. Both of these reflect recommendations for individuals with MFS, with the former being found in disease-specific support group materials (Marfan Trust, 2019) and the latter in medical guidelines (Ades and Group, 2007).

This family felt that emotional and financial stress contributed to the severity of aortic dilatation in MFS. Indeed emotional stress has been associated with spontaneous coronary artery dissection, with patients perceiving that it precipitated the event (Saw et al., 2014, 2017). Other studies found that stress and worry were perceived risk factors for genetic diseases generally (Cohen et al., 1998; Parrott et al., 2004; Shaw and Hurst, 2008), though there is currently no scientific evidence which aligns with this belief.

Most participants believed that other genes could be modifying the phenotype in MFS, and some speculated that the genetic fitness of the other parent played a role, though they admitted that identifying a good genetic match would be challenging. Other factors which participants felt could influence severity included environmental factors such as “germs,” chemicals and pollution. The concept that viruses or other infectious diseases could cause genetic conditions has been reported previously (Cohen et al., 1998; Shaw and Hurst, 2008), though usually the infectious agent is blamed for causing the disease, not initiating heritable genetic change *per se*. The quotations included provide further evidence of people’s ability to hold multiple beliefs (Cohen et al., 1998; Klitzman, 2010) in order to restore their belief that the world is “coherent, cohesive and predictable” (Shiloh et al., 2002, pg 373).

Chance or bad luck were not embraced by this family as possible explanations for phenotypic variability, though some felt it played a role in the inheritance of the *FBN1* mutation and disease causation. A previous study on causal attribution in MFS found that 54% endorsed chance as having a causative role (Peters et al., 2001). The majority of those people were the first case in the family and thus had a *de novo* mutation. However, a third of individuals from families with an inherited mutation also thought that chance played a role (Peters et al., 2001), similar to the proportion observed in this study.

The majority of individuals in this family rejected the idea that spirituality moderated the severity of disease. This did not appear to reflect a lack of faith but rather an unwillingness to blame the spirits or higher powers. One person felt that MFS could have been caused by upsetting the spirits, a common causal attribution for genetic diseases and birth defects generally (Cohen et al., 1998; Shaw and Hurst, 2008; Klitzman, 2010; Abad et al., 2014). In qualitative studies of causal attributions for cancer in Indigenous Australians specifically, disease etiology is often associated with the spiritual world of curses (Prior, 2005, 2006, 2009; McGrath et al., 2006); this has also been observed in Native Americans (Burhansstipanov et al., 1999) and African Americans (Cohen et al., 1998). Of note, a causal attribution study undertaken in the Philippines found that MFS was thought to occur when a mythical tree giant impregnated a mother so the offspring had his physical characteristics - i.e., tall stature, long limbs, and arachnodactyly (long fingers) (Abad et al., 2014). The idea that spirits can be protective is not well captured in qualitative research of Australian Aboriginal people. However, when discussing spirituality generally in these interviews, another participant commented on the spirits offering assistance, while another participant points out that the spiritual tie to the land could compromise access to quality healthcare and services. The psychological and spiritual challenge of leaving home in order to be evaluated and treated has been described by rurally located Indigenous Australians (McGrath et al., 2006; McGrath, 2007; Anderson et al., 2008; Prior, 2009). The desire for a culture-centered outreach approach has been documented (Mincham et al., 2003; **?**; Askew et al., 2014) and, to that end, there have been pilot programs initiated in some states in Australia (Hayman et al., 2014; Askew et al., 2016; Davy et al., 2016).

This study has a number of limitations. Qualitative studies do not involve probability sampling, and hence the findings reported are not generalizable to all Australian Aboriginal families with MFS. Members of a single family were assessed, and views may have been more heterogeneous had additional families been interviewed. Also, those who elected not to participate may have had different lived experiences than those who did participate. Furthermore, there was a lack of geographic and educational diversity in this family. A relatively small number of individuals opted to participate in this study. Nonetheless data saturation was achieved, and thus the small size of the sample is not seen as a limitation of this study (Patton, 1990). The retrospective, reflective nature of the interviews means that there may have been some recall bias. In hindsight, it is possible that the wording of some of the questions was not optimal; for example, it is not clear that all participants fully understood the nuance of factors causing disease as opposed to factors contributing to disease severity.

This qualitative study assessed the range of beliefs in relation to MFS, and future quantitative studies should assess the extent to which these beliefs are endorsed. This is the first study to explore causal attribution in any genetic condition in the Australian Aboriginal population; it would be interesting to conduct further qualitative research in the area of genetics in this population in the future. The concept of events that give rise to mutations is worth further exploration. If sample size permitted, such qualitative research would ideally be combined with quantitative measures (e.g., use of the Revised Illness Perceptions Questionnaire (Moss-Morris et al., 2002), which has not yet been administered to an Aboriginal Australian population). As causal perceptions affect coping, adjustment and many health behaviors, a better understanding in the Indigenous Australian population would be beneficial as genetic testing is increasingly used in a wide variety of healthcare settings.

## Data Availability Statement

The datasets for this article are not publicly available due to concerns regarding participant/patient anonymity. Requests to access the datasets should be directed to the corresponding author.

## Ethics Statement

The studies involving human participants were reviewed and approved by University of Queensland Human Research Ethics Committee. The patients/participants provided their written informed consent to participate in this study.

## Author Contributions

AM-L designed the study, with guidance from BM, ED, and MB. JW and MW provided the introductions to members of the large family with Marfan syndrome and traveled with AM-L to remote towns in Queensland to maximize the chances of participation. AM-L conducted the analysis of the data with oversight from BM and also read and coded a subset of the transcripts in parallel and any discrepancies were discussed and reconciled.

## Conflict of Interest

BM has a remunerated consultant role with the company AstraZeneca with respect to an unrelated project.

The remaining authors declare that the research was conducted in the absence of any commercial or financial relationships that could be construed as a potential conflict of interest.
